# Soybean Genomics: Developments through the Use of Cultivar “Forrest”

**DOI:** 10.1155/2008/793158

**Published:** 2008-04-23

**Authors:** David A. Lightfoot

**Affiliations:** ^1^Department of Plant Soil and General Agriculture, Center for Excellence, The Illinois Soybean Center, Southern Illinois University at Carbondale, Carbondale, IL 62901-4415, USA

## Abstract

Legume crops are particularly important due to their ability to support symbiotic nitrogen fixation, a key to sustainable crop production and reduced carbon emissions. Soybean (*Glycine max*) has a special position as a major source of increased protein and oil production in the common grass-legume rotation. The cultivar “Forrest” has saved US growers billions of dollars in crop losses due to resistances programmed into the genome. Moreover, since Forrest grows well in the north-south transition zone, breeders have used this cultivar as a bridge between the southern and northern US gene pools. Investment in Forrest genomics resulted in the development of the following research tools: (i) a genetic map, (ii) three RIL populations (96 > n > 975), (iii) ∼200 NILs, (iv) 115 220 BACs and BIBACs, (v) a physical map, (vi) 4 different minimum tiling path (MTP) sets, (vii) 25 123 BAC end sequences (BESs) that encompass 18.5 Mbp spaced out from the MTPs, and 2 000 microsatellite markers within them (viii) a map of 2408 regions each found at a single position in the genome and 2104 regions found in 2 or 4 similar copies at different genomic locations (each of >150 kbp), (ix) a map of homoeologous regions among both sets of regions, (x) a set of transcript abundance measurements that address biotic stress resistance, (xi) methods for transformation, (xii) methods for RNAi, (xiii) a TILLING resource for directed mutant isolation, and (xiv) analyses of conserved synteny with other sequenced genomes. The SoyGD portal at sprovides access to the data. To date these resources assisted in the genomic analysis of soybean nodulation and disease resistance. This review summarizes the resources and their uses.

## 1. INTRODUCTION

The soybean cultivar “Forrest,” a product of a USDA breeding program, represents a determinate, Southern germplasm [1]. It was the first cultivar to possess soybean cyst nematode (SCN) resistance associated with high yield, and is believed to have played a key role in saving billions of US dollars during 1970s and 1980s that would have otherwise been lost, either due to SCN or due to the poor agronomic performance of earlier SCN resistant cultivars (see [2] and references therein). Forrest was an important parent of modern cultivars, “Hartwig,” “Ina” and many others that have an improved SCN resistance gene from PI437654 introgressed into their genome [3–5]. Forrest was also central to an understanding of the genetics of resistance to sudden death syndrome, an important new disease of soybean [6–9].

**Figure 1:**
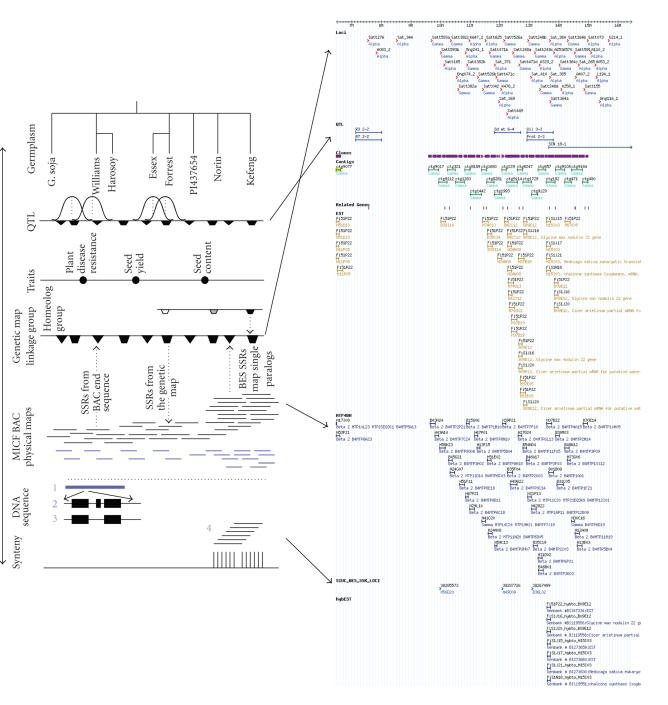
Soybean genomic resources and products schematic for Forrest (A) compared to the SoyGD representation (B). Panel A. Germplasm that are exemplars of soybean genetic diversity are shown. Selected germplasm encompass in mapped QTL a wide variety of traits placed on the composite genetic map. BAC libraries exist for many of the germplasm sources. Forrest BACs (shown in black) form the basis of an MICF physical map with 6-fold coverage. A region of conserved duplication (12-fold coverage) is shown on the right of the figure. In this region, fingerprinted clones from two homoeologous linkage groups coalesce. Genetic markers identified in, or derived from, BAC end sequences (BESs) will separate some of the duplicated conserved regions. Genetic markers anchored from map to BAC are of little use in conserved duplicated regions. BACs from diverse germplasm are shown as blue bars. There are 3 levels of DNA sequence envisioned. At level 1, BESs provide a sequence every 10–15 kbp with which to identify gene rich regions for later complete sequence determination (level 2). Arrayed BAC end sequences will be used to identify conserved syntenic regions in the genomes of model plant species. This information will also separate some of the duplicated conserved regions in soybean. Panel B. Shown are the chromosome (cursor), DNA markers (top row of features, red); QTL in the region (second row, blue); coalesced clones (purple) comprising the anchored contigs (third row, green); BAC end sequences (fourth row black); BESs encoding gene fragments (fifth row, puce); EST hybridizations to MTP2BH (sixth row gold); MTP4BH clones (seventh row, dark blue); BESs-derived SSR (eight row, green).

Forrest is also one of the two cultivars (the other being “Williams 82”), providing the majority of genomic tools for soybean, available in USA (Figure 1) [10, 11]. These two cultivars provide models for soybean genomics research in the same way as are the cultivars *Col* and *Ler* in *Arabidopsis thaliana* or Mo17 and B73 in *Zea mays*. However, since the genomics of “Williams 82” was recently reviewed [11], its inclusion in this article would be repetitive. The other cultivars, which represent the worldwide germplasm variation for soybean genomics, include the following: (i) “Noir 1,” a Korean plant introduction (PI) [12], (ii) “Misuzudaizu,” a Japanese cultivar [13], and (iii) “Suinong14,” a Chinese cultivar [14]. The soybean community is committed to advance the genomics of all these cultivars, which have been used in the past as resources for genomics research. However, the intent of this review is to present an overview of the genomic resources derived from Forrest; these genomics resources enable a wide range of analyses that address several fundamental questions, like the following: (i) what is the source of genetic variation in soybean improvement? [15]; (ii) what is the role of variation in regions of genome duplication in paleopolyploid species? [16]; (iii) how does the nodulation of legumes work? [17]; (iv) why are protein and oil contents of seed inversely related? [18, 19]; (v) why are seed yield and disease resistance so hard to combine? [4, 5, 15, 20]; (vi) why is seed isoflavone content limited below 6 mg/kg? [18, 21–24]; (vii) how does partial resistance to disease work [6–9, 18]? It is believed that the development and use of genomics tools derived from Forrest will help soybean researchers to provide answers to these questions.

## 2. GENETIC VARIATION BETWEEN FORREST AND OTHER CULTIVARS

**Figure 2:**
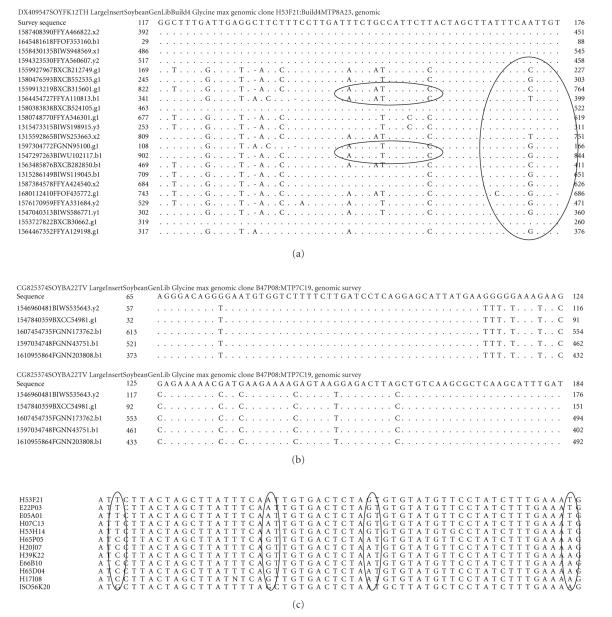
Comparison of MegaBlast analysis of an unduplicated region and a twice duplicated region as inferred by the fingerprint physical map (a). Analysis of the BESs from H53F21 in quadruplicated contig 9077. These BESs contained a very common repeat with 400 copies per haploid genome. Sequence analysis supported the inference of four copies of the region per haploid genome made from BAC fingerprint data (a). MegaBlast of H53F21 (Build4MTP8A23, gi89261445) against 7.3 million reads with repeated masking gave 7 identical matches among 24 homoeologous sequences. Cluster 1 was composed of traces ending in …822,…160,…569,…607,…662,…749, and …105 that shared A at position 172 (circled). Homoeolog specific variations (polymorphisms) were evident among the 4 clusters inferred. Cluster 2 was composed of clones ending in 749, 850, and 601 that shared C at position 172. Cluster 3 was composed of clones ending in 100, 117, and 535 that shared G at position 172. Cluster 4 also had G at that position. TreeCluster analysis showed the most similar homoeologs clustered into 4 separate sets as expected for regions duplicated twice (circled) (b). Analysis of the BESs from B47P08 in contig 321 from an unduplicated region. Sequence analysis supported the inferrence of an unduplicated region made from fingerprints at 90% sequence identity (c). The sequences found among BACs resequenced from contig 9077 showing a set of SNHs (HSVs) separated two groups of the four inferred to be present: the A cluster and the G cluster (adapted from [29]).

**Figure 3:**
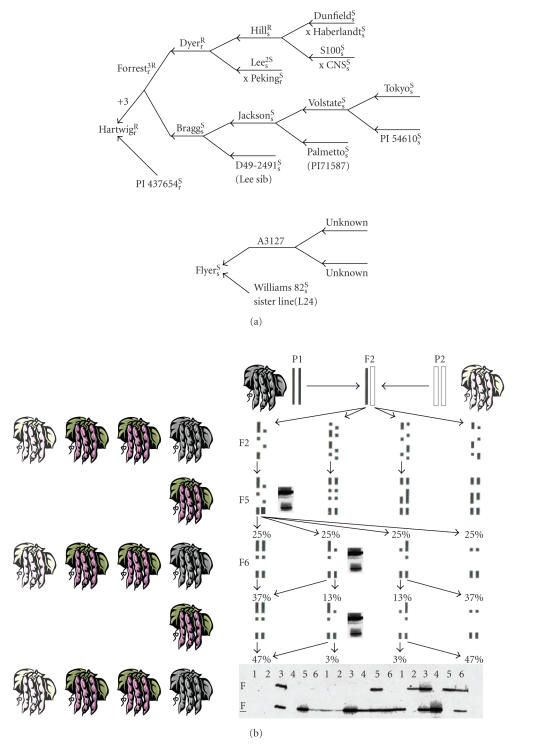
Genetic systems used with Forrest germplasm and the inbred soybean crop (a). The ancestry of Forrest and Hartwig showing the known cultivars that were crossed and the relationship between Flyer and Williams 82 (b). A diagram showing how NILs derived from RILs fix most loci but allow the continued segregation of heterozygous regions in inbred crops like soybean. The effect is to Mendelize a few of the loci contributing to QT while causing the majority to be fixed. A dark pod parent was crossed with a light colored pod parent; the F1 heterozygous type (shown as purple pods) was selfed; and F2 progeny was advanced to the F5 by selfing. A heterozygous plant at any time or heterogeneous RIL at F_5:7_ or later identified is shown as purple pods. Single plants are extracted and seed increased. NILs that result may fix the heterogeneous region to the parent 1 allele, the parent 2 allele, or are still heterogeneous. Occasionally heterozygous plants are found within some heterogeneous NILs even at the F_5:15_ and the progeny of such plants can be used to find new recombination events. Shown are the results with Satt309 and NIL11 plant 3 and eighteen of the progeny collected from it (adapted from [40]).

An important question that received the attention of soybean researchers in the past is how much sequence variation one can expect between Forrest and other cultivars, if many are to be sequenced. This variation is extensive (about 1 bp difference per 100–300 bp), when judged by using the criteria like the following: (i) the coefficient of parentage [25], (ii) the number of shared RFLP bands [26], (iii) polymorphism among microsatellite markers [27], and (iv) DNA sequence comparisons (Figure 2). In soybean, the degree of linkage disequilibria among loci is high, extending over distances that range from 50 kbp to 150 kbp [28]. Few meioses have occurred within these regions to reshuffle the gene or DNA sequences, because soybean is largely an inbreeding crop. In recent times, only seven or eight crosses have been made, starting from the time when the PIs were collected to the development of most modern US cultivars (Figure 3). Therefore, in different parts of the genome, LD encompasses large segments and sets of genes.

### 2.1. The Essex × Forrest population

A soybean recombinant inbred line (RIL) mapping population (Reg. no. MP-2, NSL 431663 MAP) involving Forrest was recently developed from the cross “Essex MAP” (PI 636326 MAP) × “Forrest MAP” (PI 636325 MAP) [10]. This RIL population was used for constructing a genetic map [9, 24, 30] that has been used extensively for an analysis of marker-trait associations [7–9, 24, 30–38]. The genetic marker data encompass thousands of polymorphic markers and tens of thousands of sequence-tagged site (STS) that were collected at SIUC by Dr. Lightfoot’s group (Table 1) [10]. The genetic maps of E × F94 will continue to be enriched [27, 39]. The registration of this population [10] has allowed public access to the population and data generated from it worldwide.

A key feature of the above mapping population is that Essex (registered in 1973 [10]) was derived from the same southern US germplasm pool to which Forrest (registered in 1972 [1]) belongs. Consequently the RILs share identity across about 25% of their genomes, the portion that was monomorphic in both of the parents (Figure 3) [25, 26]. Further, the two cultivars were selected under similar conditions and, therefore, appear rather similar in most environments [6–10, 15–20, 30–38]. However, detailed records of maturity dates are important, since even a single day variation in maturity may influence the results of QTL analysis for many other traits [10, 41]. Since morphological and developmental traits differ very little in the population, the RILs have been used extensively to map those genes which control biochemical and physiological traits (Table 2). For example, the parents of the mapping population differ by resistance traits, which exhibit both qualitative and quantitative inheritance (Table 3).

A major limitation in using E × F population in genomics research is the small population size (*n* = 100) that could preclude fine mapping [10]. To overcome this problem, populations of near isogeneic lines (NILs; *n* = 40; Figure 3) were developed from each RIL [10, 37, 38, 43]. The NIL populations are listed in Table 1. The residual heterozygosis present in the F5 seed was largely fixed and captured in these NILs. The heterogeneity across the RILs has been measured to be 8%, which is more than the 6.25% expected among F5 lines [7, 24]. That increased heterogeneity appears to be caused by selection, since rare heterozygous plants still exist in some RILs and NILs [37, 38, 40]. Each locus that segregates in the RIL population is expected to segregate in about eight NIL populations. Therefore, each region in the genome will be segregating in about 420 lines (100 + 8 × 40), quite sufficient to create fine maps of 0.25 cM resolution (Table 4). A 0.25 cM interval represents 25–100 kbp on the physical map [16], sufficient for candidate gene identification [37, 38].

Consequent to the development of the NILs, the E × F population was used to study the genetics of a large number of quantitative traits (QTs), leading to the identification of quantitative trait loci (QTL; Table 2) underlying more than seventy different traits [24, 39, 40, 42, 44–46]. Biochemical and physiological traits included resistance to soybean sudden death syndrome (SDS) [caused by *Fusarium virguliforme*] in the US and Argentina, resistance to soybean cyst nematode (SCN; *Heterodera glycine* Ichinohe), seed yield, seed quality traits, agronomic traits, water use efficiency, manganese toxicity, aluminum toxicity, partial resistance to *Phytophthora sojae*, and insect herbivory. However, new opportunities abound because dozens of traits for resistance to pests and pathogens segregate in the population but were not yet mapped [10]. Further, the concentrations of many secondary metabolites among lines vary widely during development and among different organs [47]. Pesticide uptake, metabolism and degradation rates also vary among lines (unpublished). Preliminary studies have shown the link between the genome, proteome, and metabolome (the interactome), which can be further explored in these segregating populations [48]. Therefore, E × F will eventually be used to map thousands of QTL for hundreds of QT.

Importantly, the NILs that have been developed from each RIL for fine mapping also allow confirmation of QTL detected in the RIL population. For instance, *cqSDS001* was assigned to a QTL confirmed by NILs derived from Ripley [49], but earlier detected through RILs derived from Flyer [50] and “Pyramid” [6, 33]. The QTL have also been renamed under the new rules for QTL adopted by the Soybean Genetic Committee in 2006 [51], as a result of which *cqRfs1*, *cqRfs2*, and *cqRfs4* were renamed as *cqSDS003, cqSDS002*, and *cqSDS004*, respectively.

The molecular linkage map, the RILs, and the NILs were used during the positional cloning of *nts1, GmNARK* [50], *Rpg1* [17, 35], *Rhg1*, [38] *Rhg4*, [52], and *Rfs2* [37]. Many opportunities for further gene isolations exist. Tables 2 and 3 list some of the known phenotypes that differ between the parents and segregate among the lines and that are candidates for gene isolation. The RIL and NIL populations provide sets of recombination events that can be used to identify the positions of genes underlying QT [10]. Since all the lines self-fertilize, the populations can be used to provide an immortal resource, if seed germination ability can be regenerated every five years. This type of resource is particularly important for soybean because the draft genome sequence will be released in April 2008 (unpublished). Combining knowledge of locus positions with a comprehensive knowledge of gene content will lead to the rapid isolation of many new and economically important genes [16].

**Figure 4:**
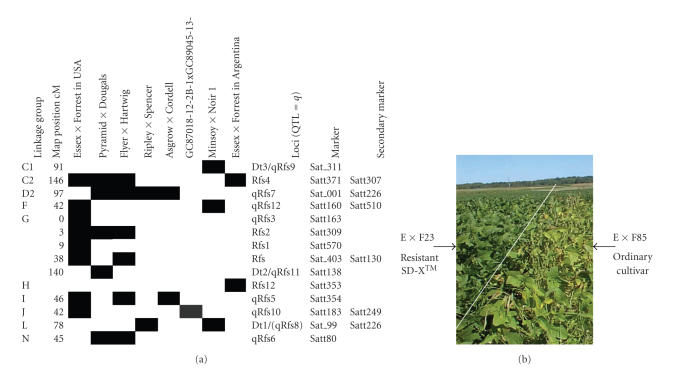
An example of the use of Forrest genomics resources for soybean germplasm improvement (a). Summary of the map locations of the known loci for resistance to SDS. A black rectangle indicates that the allele is segregating in that population. Nonsegregating alleles may be either fixed to the resistance or susceptibility forms (b). An example of quantitative variation for disease resistance identified in lines derived from Forrest. The resistant line RIL23, left of the line, has beneficial alleles for six QTL for resistance to *Fusarium virguliforme*. The leaf scorch associated with the fungal infection is evident in the neighboring RIL80 to the right of the white line.

Selected lines from the E × F population that contrast for mapped QTL were also used for a variety of studies including the following: (i) to validate assays of pathogenicity [32, 53–55], (ii) to examine the effects of resistance genes on gene expression [34, 56, 57], (iii) to analyze components of drought tolerance [24, 31, 36, 42, 46, 58], (iv) to validate methods of marker assisted selection [6, 31, 59–62], and (v) to provide for germplasm releases (Figure 4) and cultivars [6, 63]. New cultivars and new methods for selection of improved soybean genotypes are among the most important spin-offs from the genomics research involving Forrest soybean. Among the selected lines, E × F78 later became LS-G96 [63] and then “Gateway 512” (Gateway Seeds, Nashville, Ill, USA). This line together with the line E × F55 was used as parents that combined moderate resistance (carrying resistance alleles at six loci) to SDS with high yield. The RIL E × F23 was released as SD-X for very high resistance to SDS [34] and good yield potential under license from Access Plant Technologies (Plymouth, Ind, USA), because it contained beneficial alleles at all eight known resistance loci. In contrast, E × F85 is susceptible to SDS as it contained no beneficial alleles at the known resistance loci. It makes a great entry for sentinel plots. For animal feed and human food, E × F52 has been used as a parent to provide very high phytoestrogen contents to progeny (unpublished), since it contained beneficial alleles at all the known loci underlying phytoestrogen content. Low phytoestrogen contents are also required for estrogen sensitive consumers; E × F89 and E × F92 were used as parents to provide parents for low phytoestrogen in the progeny (unpublished).

### 2.2. Related populations flyer by hartwig (F × H) and Resnik by Hartwig (R × H)

The F × H and R × H populations are integrated with E × F96 [10], since Forrest was the recurrent parent used to develop Hartwig (Figure 3) [62] and Essex shares many alleles with the Flyer and Resnik [15, 27]. Flyer and Resnik were sister lines derived from a cross between a Williams 82 sister line and a commercial cultivar [64]. The F × H has 92 RILs and R × H has 952 RILs that have been used to confirm QTL detected in E × F96 and for fine mapping of these QTL [4, 5, 15, 50, 52]. Flyer and Resnik each contains many genes conferring resistance against *P. sojae*. Both these populations can be used to map genes underlying additional biochemical, physiological, and some agronomic traits that include the following: (i) resistance against *Phytophthora* root rot, soybean sudden death syndrome (SDS) caused by *F. virguliforme* and soybean cyst nematode (SCN), *Heterodera glycine* Ichinohe, (ii) seed yield [15, 50, 52], and (iii) seed quality traits. These RILs were also used to develop SSR markers that anchor contigs and sequence scaffolds (http://soybeangenome.siu.edu/) to the physical map [27].

## 3. PHENOTYPIC VARIATION BETWEEN FORREST AND OTHER CULTIVARS

One major limitation using the resources based on Forrest was the low amount of genetic variation detected in the populations based on this cultivar [65]. The implication was that the alleles detected in E × F would not be weaker variants of the major gene effects found in weedy plant introductions (PIs). It was hypothesized that, instead, the loci detected in the E × F population and in the material derived from this population perhaps represented other gene systems of lower hierarchical position and therefore lower value. Consideration of a few examples of the locations of QTL underlying phenotypic variation between Forrest and other cultivars has been informative regarding this issue. The results to date all infer that the alleles underlying QTs in Forrest are variations in the same genes as the PI alleles, if weaker in effects on QTs.

### 3.1. The genetics of phytoestrogen content

The phytoestrogen content of soybeans seed mainly consists of daidzein (60%) and genistein (∼30%) with small proportion of glycitein (∼10%) [66]. Analysis of germplasm and elite cultivars (18, 21–24, 67–69) indicated that phytoestrogen concentrations in some elite cultivars (∼2 mg/kg) were higher than those in many of the ancestors of cultivated soybean (∼1 mg/kg). Phytoestrogen content and profile varied with environment (year and location effect) and genotype. However, the final seed content was largely controlled by the genotype (40–60% of the variation) and is controlled by a set of about 6–12 loci [18, 24, 67]. If the content of each phytoestrogen component was controlled independently, improvements in content by genetic selection should be possible. For instance, raising glycitein content to the same amount as that of daidzein could double the total phytoestrogen content. However, because heritability of phytoestrogen content is moderate at about 40–60%, direct selection (without DNA markers) has not been very effective. Through marker-assisted selection (MAS), the phytoestrogen amounts were raised to 3.6 mg/kg, well above the amounts found in elite cultivars or weedy PIs. Here, the variation programmed by the alleles segregating in E × F population was greater than that among the entire germplasm collection.

Recently, crosses have been made betweenlines from southern Illinois and Canada having the highest phytoestrogen contents [23] and, separately, the lines having the lowest phytoestrogen content [67]. MAS exercised in the segregating populations (at F4 in 2007) should lead to improvement in phytoestrogen content. Opportunities for collaborative studies exist with sets of RILs in maturity groups that are not adapted to be grown in southern Illinois or Canada.

### 3.2. The genetics of seed yield, protein and oil content

The overall average increase of 1-2% per year in soybean yield witnessed during 1960–1999 was only half the yield advances achieved in corn and other out crossing crops, where genetic diversity was not limiting [68]. As one would expect, there are hundreds of loci controlling yield in soybean [69]. In view of this, half of the yield loci detected in E × F population were those which were earlier detected in other crosses [24]. These loci could each boost seed yield by 0.2 Mg/Ha. In contrast, substantial gains (0.9–1.1 Mg/Ha) can be made in soybean yield by identifying unique alleles in weedy PIs and introgressions into elite cultivars [70]. The nature of the genes altering seed yield will be an interesting product from fine map analysis and positional cloning.

The major components ofsoybean seed yield include the following: (i) protein (∼40%), (ii) oil (∼20%), (iii) structural carbohydrates (∼6%), (iv) water (∼13%), (v) soluble carbohydrates (∼14%), and (vi) other metabolites (∼7%) [71]. Metabolic changes during development driven by gene expression underlie the seed composition and yield [72]. Seed yield and composition are under polygenic control with different genes active at different stages of seed development. Seed traits are also associated with significant genotype × environment (G × E) interactions as observed in E × F population (see [15, 18, 19]). Again, the G × E interactions significantly reduce the effectiveness of visual selection based on the phenotype alone.

At harvest, seed protein content is inversely related to seed oil content and seed yield in E × F population [18, 19] as also in other germplasm (see [68]). While some loci are implicated in all the three traits, there are others which influence only one or two of the three traits. Several QTL underlying soybean yield, protein, and oil content have been mapped in both the E × F and the F × H RIL populations [5, 18]. They do correspond with loci detected in crosses between high protein weedy types and low protein adapted cultivars. Three QTL on linkage groups A1, A2 and linkage group E have been fine-mapped and localized within 0.25 cM using substitution mapping to identify the underlying genes. Isolation of these genes will partly explain the molecular basis of the genetic control of yield and its component traits. However, a danger here is that because different genes are active at different stages of seed development, one would generally map only a composite trait, based on a mean of the action of several loci. Isolation of genes by position would not be successful in this circumstance.

### 3.3. The genetics of *Phytophthora* root rot resistance

The annual soybean yield loss suffered from the root and stem rot disease caused by the oomycete pathogen, *Phytophthora sojae* is valued at about $273 million in the US [73]. Monogenic resistance due to a series of *Rps* genes has been providing a reasonable protection to the soybean crop against the pathogen over the last four decades [74]. Several mapped *Rps*-genes are known to occur in Flyer and Resnik [50, 64]. Partial, rate-reducing resistance to many races of *P. sojae* is found also in Forrest, Essex, and Hartwig. The loci providing this partial resistance were not mapped by 2007.

### 3.4. The genetics of SCN resistance

Soybean cyst nematodes (*Heterodera* *glycines* I.) are the most damaging pests of soybean worldwide [73]. Development of resistant cultivars is the only viable control measure [75]. Resistance genes have been found to be located on 17 of the 20 chromosomes by 2007. A combination of recessive genes is necessary to provide resistance against SCN populations because many are known to be capable of overcoming all known single resistance genes. SCN populations can be classified into 16 broad races or up to 1024 biotypes (HG Types) [76] based on the host responses of 8 weedy indicator lines. SCN resistance in many other adapted and weedy cultivars [9, 31] shared the same loci underlying bigeneic inheritance in E × F [20]. The E × F population was used to isolate candidate genes for those two loci (*rhg1* and *Rhg4* ; Table 4) that control resistance against SCN race 3 (HG Type 0). Alleles of the candidate genes were identified in many PIs through association studies [38, 77]. Paralogs of both these genes were found at new locations in BAC libraries and whole genome shotgun (WGS) sequences [78, 79]. They appear to be part of multigene families showing homoeology and intragenomic conserved synteny.

Three cultivars including Peking, PI437654, and Hartwig encoded 2–4 additional genes that provide additional resistances to SCN [52, 80, 81]. Peking has alleles for resistance to races 1 and 5 that were not transferred to Forrest [20]. Hartwig and PI437654 have complete resistance against all races of SCN except race 0, HG Type 1.2.3.4.5.6.7.8. The location of SCN resistance loci in F × H and R × H agreed with those found in crosses between PIs and adapted germplasm [81, 82]. Therefore, the resistance to SCN traits that are introgressed from PIs to Forrest-based germplasm is useful and the underlying genes can be isolated from Forrest.

### 3.5. The genetics of SDS resistance

Soybean sudden death syndrome caused by *Fusarium virguliforme* (e.g., *solani* f. sp. *glycines*) is among the most damaging syndrome of diseases affecting soybean in the US and worldwide [73]. The syndrome is composed of a root rot disease and a leaf scorch disease [53]. Development of resistant cultivars is the only viable control measure. Twelve resistance loci have now been found on 8 chromosomes (Figure 4), eight segregate in E × F [24, 44] and two additional loci segregate in F × H [5, 50]. A combination of loci is needed to provide resistance to both root rot (2 or more loci) and leaf scorch (all loci). Loci for resistance to SDS were named *Rfs* to *Rfs11* [39]. Using NILs (Table 4), a set of candidate genes for the *Rfs2* locus were identified [37]. Among these genes, a receptor like kinase [38] and a laccase [83] are being tested for their ability to provide resistance following transformation and mutation (unpublished). However, the presence of a pair of syntenic genes on linkage group O with similar DNA sequences (84%) and encoding nearly identical amino acid sequences (98%) complicates the analysis following reverse genetics approach.

One of the two loci underlying root rot resistance is encoded in the DNA sequence around marker OI03_514_ that lies between AFLP derived SCARs, CGG5, and CTA13 on linkage group G [37]. However, the root rot resistance locus (Table 4) lay in a region not well represented among BAC libraries [84, 85], so that the gene isolation was delayed until the local genome sequence could be assembled. Transcript analysis showed that the fungus attempts to prevent gene transcription in the target roots [34, 55, 56]. Resistant cultivars prevent the poisoning of transcription by inducing stress and defense genes that produce fungicidal metabolites within 2 days of contact with the fungus. However, the induced genes do not appear to map to the loci that control the SDS resistance response [57]. Instead, genes of a higher hierarchical position in the interactome were found in this interval (unpublished). One of these genes is expected to underlie root resistance to SDS.

For the fungus, *F. virguliforme* causing SDS, no races are known so far in the US [86]. When lines from E × F have been used to look at variations in pathogenicity between strains, no convincing evidence for a host differential response was observed (unpublished). However, different Fusarium species that are capable of causing SDS are found in South America [86]. E × F was planted in Argentina since 2004, and it was shown that the SDS pathogen(s) invoked responses that mapped to different resistance loci [39]. Therefore, the fungus does have the potential to form races that vary in their pathogenicity. Hence, soybean breeders should be cautious in using the available resistance genes and should realize that stacking of all the twelve genes for full resistance would not be wise because it would select for mutants in the pathogen populations that could lead to the development of races.

In conclusion, a variety of approaches including QTL analysis, fine map development for some loci, and analysis of isolated genes have revealed that the alleles detected in E × F are variants of the same major genes found in weedy plant introductions (PIs) [5, 24, 41, 53]. Only few loci detected in the E × F population and in the other materials derived from this cross seem to represent other gene systems at a lower hierarchical position [57]. Identification of the lower tier of genetic control may require intercrosses among NILs or assays that relate to development, time, position, or cell type.

## 4. STRUCTURAL GENOMICS RESOURCES

Soybean (*Glycine max* L. Merr.) has a genome size of 1115 Mbp/1C [87]. The soybean genome is the product of a diploid ancestor (*n* = 11), that underwent aneuploid loss (*n* = 10), allo- and autopolyploidization events separated by millions of years (*n* = 40) with reversion to a lower ploidy after one of those two events (*n* = 20) [88]. Evidence that two genome duplications occurred, 40–50 MYA and 8–10 MYA, was supported by RFLP analysis suggesting 4–8 homoeologous loci for most probes [89] and discontinuous variation among paralagous EST sequences [90–92]. Even PCR-based markers that can amplify single loci from genomic DNA amplify multiple amplicons from BAC pool DNA (Figure 2). The duplicated regions have been segmented and reshuffled after the polyploidization events [16, 93–95].

**Figure 5:**
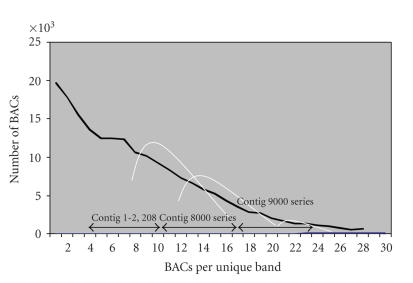
Quality estimate for the physical maps build 4 showing measurements of BAC clones per unique band. Three sets of distributions were inferred, representing the diverged DNA and the conserved DNA following the two genome duplications (shown as white lines). The 2208 single copy contigs (labeled 1–3500 after merges and splits) encompassed diverged DNA and are each inferred to contain clones from a single region. Contigs in the 8000 series are inferred to contain clones from two homoeologous regions. Contigs in the 9000 series are inferred to contain clones from four homoeologous regions. Clearly, some contigs in each set will be missplaced, hybrid contigs will occur, and ranges will overlap.

Recently, a systematic measurement of DNA sequence divergence between homoeologous regions was made possible by comparing Forrest BAC end sequences with 7 million reads from the WGS sequences of Williams 82 [29, 93]. MegaBlast searches distinguished some regions, resolving up to 10% nonidentity between homoeologs over a 60 bp window (Figure 2). This implied that significant sequence divergence has occurred at about half the loci tested, as predicted from the gene-family size distribution observed in the physical map [57] (Figure 5). Conversely, highly conserved regions (>90% identity) exceeding about 150 kbp (the size of a large insert clone) have been inferred in certain regions [29]. Within these regions, 2 or 4 homoeologs can be distinguished by single nucleotide variants that correspond to the duplicated regions of a paleopolyploid genome or recently polyploid genome. These variants have been described as single nucleotide polymorphisms among homoeologs (SNHs) [93] though they are commonly called homoeologous sequence variants (HSVs) (see, e.g., [91]).

Overlain on the segmented regions found in 2 or 4 copies, the soybean genome is a composite of dispersed and contiguous euchromatic regions [88]. The short arms of four chromosomes are entirely heterochromatic, but in the remaining 16 chromosomes with potentially gene rich euchromatic arms, the heterochromatin is restricted to pericentromeric regions. Euchromatin represents 64% of the soybean genome, with a range of 40–85% on an individual chromosome. Due to these features and the following other reasons, analysis of soybean genome has been a challenge: (i) large genome size, (ii) serial duplication of regions, (iii) small proportion of unique DNA, and (iv) highly conserved repeated DNA. One reasonable prediction would be that many of the duplicated regions would be silenced in heterochromatin. However, a comparison of the genetic map and physical map [93–95] has shown that duplicated segments are neither clustered nor restricted to heterochromatic arms. Further, the gene-rich islands are not separate from the duplicated regions. Therefore, new models to explain gene regulation that include duplicated conditions must be developed. Lessons learned from this exercise will help in the analysis of some legume and many dicotyledonous crop genomes, where genome duplication is believed to have often accompanied speciation. Breeders, who develop new cultivars through selection from the available variation within a cultivar, will also utilize this information and will develop new selection methods through an understanding of the effects and benefits of partial, segmented, genome duplication.

### 4.1. BAC libraries and physical maps

Construction of fingerprint-based physical maps in soybean relied on the availability of deep-coverage high-quality large insert genomic libraries, and a number of such public sector large insert libraries are available in four different plasmid vectors, providing >45-fold genome coverage. BAC libraries are available not only for Forrest and PI437654, but also for some *G. soja* PIs and the wild relatives of *G. max* [84, 85, 96, 97]. Among these libraries, there are three “Forrest” BAC libraries [84, 85], available in two different plasmid vectors with different *ori*s and different selectable markers (Table 5). Despite the availability of these rich BAC resources, there are still a few regions of the genome that are not well represented across the above set of BAC libraries. New libraries without involving restriction digestion may help solve this problem (unpublished).

A double-digest-based physical map for the soybean genome is now nearing completion. For this purpose, soybean BACs from five libraries belonging to three cultivars were fingerprinted and assembled [98] using a moderate information content fingerprint method (MICF) and FPC. The available BACs presently include 1182 Faribault BACs (∼130 kbp, *Eco*RI inserts, 0.125x), 860 Williams 82 BACs (∼130 kbp, *Hind*III inserts, 0.1x) and 78 001 Forrest BACs that were selected from the three libraries (125–157 kbp *Eco*RI, *Hin*dIII, and *Bam*HI inserts, 9x). Cultivar sequence variation did not appear to cause incorrect binning of BACs by FPC. However, the first release (build 3) [98] had many problems (Table 6), since many individual contigs appeared to contain noncontiguous genomic regions, and in some cases, different contigs contained the same region of the genome. Also, the available set of contigs encompassed a space that was 300 Mbp more than the size of the soybean genome. Clone contamination caused many of these problems, so that new methods to identify and eliminate contaminated clones were developed [99].

**Figure 6:**
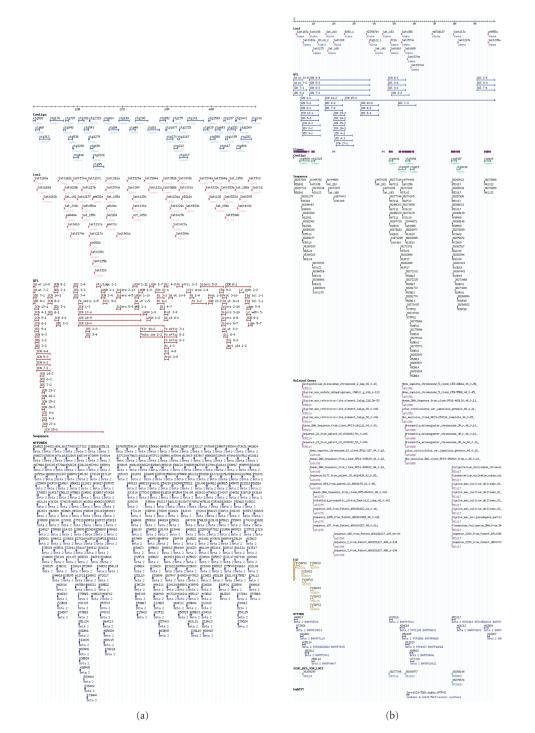
*Description of chromosome 18 resources at SoyGD (a).* The current GMOD representation of 50 Mbp of the 51.5 Mbp chromosome 18 (linkage group G) in SoyGD (a). shows the build 3 version of the chromosome (cursor), anchored contigs (top row, blue), DNA markers (second row of features, red), QTL in the region (third row, burgundy), MTP2 clones (B, H, and E fourth row, dark blue). Not shown here were BAC clones, ESTs, BAC end sequences, and gene models (b) shows the build 4 representation of 10 Mbp of the 51.5 Mbp chromosome 18 in SoyGD. Shown are the chromosome (cursor), DNA markers (top row of features, red); QTL in the region (second row, blue); coalesced clones (purple) comprising the anchored contigs (third row, green); BAC end sequences (fourth row black); BESs encoding gene fragments (fifth row, puce); EST hybridizations to MTP2BH (sixth row gold); MTP4BH clones (seventh row, dark blue); BESs derived SSR (eighth row, green); EST hybridizations inferred on build 4 from clones also in MTP2BH (ninth row, blue); WGS trace file matches from MegaBlast (tenth and last row, light blue). It is recommended for readers to visit updated site http://bioinformatics.siu.edu/ to see a full detailed color version and a build 5 view. The gaps between contigs will be filled in build 5 by contig merges suggested by BESs-SSRs and contig end overlap data.

Subsequently, the publicly available soybean BAC fingerprint database was used to create build 4 [16] with the following specific aims: (i) to increase the number of genetic markers in the map, (ii) to reduce the frequency of clone contamination, (iii) to rebuild the physical map at high stringency, (iv) to examine clone density per contig, and (v) to examine the effectiveness of the generic genome browser in representing duplicated homoeologous regions (Table 6). Clones suspected of contamination were listed, fingerprints were examined, and contaminated clones removed from the FPC database. Many (7134 about 10%) well-to-well contaminated clones were removed from the fingerprint database. The edited database produced 2854 contigs and encompassed 1050 Mbp. In addition, homoeologous regions that might cause separate contigs to coalesce were detected in several ways. First, contigs with high clone density (23%) were inferred to represent two copy (240) or four copy (406) conserved genomic regions per haploid genome (Table 6). If the polyploid regions could all be split using HSVs (Figure 1) [29], there would be 1624 regions with two copies and 480 regions with four copies in the soybean genome. A second proof of this genome structure was that pairs of separate contigs that contained the same marker anchors (69%) were inferred to represent homoeologous but diverged genomic regions (Figure 6) [16]. A third proof came from EST hybridizations to BAC libraries where gene families with 1, 2, 4, and 8 members were more common than those with 3 or 5 members [57]. Finally, similarity search within the whole genome sequence at 90% similarity showed that the sequences that map to the contigs with duplicated regions do have homoeologs in the sequence, whereas sequences from single copy regions do not (Figure 2) [29, 93].

To deal with duplicated regions, SoyGD was adapted to distinguish homoeologous regions by showing each contig at all potential anchor points, spread laterally, rather than as overlapping [16]. Therefore, it should be realized that the genes in such regions have duplicates in other regions of the genome (Figure 6). This information will prove useful in future for gene isolation by positional cloning following a reverse genetics approach, where aneupleurotic pathways regularly cause wide-spread failures [100–102] due to inability to predict phenotypes reliably.

In build 5, DNA sequence scaffolds (unpublished) have been used to cluster groups of neighboring contigs. This, however, does not solve the problems faced due to genome duplication. In many cases, (60–80%), homoeologous variants may help separation of coalesced regions [29], but this would require BESs for every fingerprinted BAC clone. In a minority of regions (20–40%), sequences longer than BESs may be needed to correctly separate BAC clones into contigs.

### 4.2. Minimum tile paths

The creation of minimally redundant tile paths (MTP) from contiguous sets of overlapping clones (contigs) in physical maps is a critical step for structural and functional genomics [95]. The first minimum tiling path (MTP) developed (from builds 2 and 3) contained 2 fold redundancy of the haploid genome (2,100 Mbp). MTP2 was 14 208 clones (mean insert size 140 kbp) that were picked from the 5597 contigs of build 2. MTP2 was constructed from three BAC libraries (BamHI (B), HindIII (H) and EcoRI (E) inserts), encompassing the contigs of build 3 that were derived from build 2 by a series of contig merges, but does not distinguish regions by degree of duplication, so that many regions are redundant. The MTP2 is used in two parts, MTP2BH and MTP2E (Table 6) because they are largely redundant and overlap each other. Also, the vectors differ in the antibiotic resistance conferred. Consequently, only the MTP2BH was used for development of EST map [57].

The third and fourth MTPs, called MTP4BH and MTP4E (Table 6), were each based on build 4 [95]. Each was selected as a single path through each of the 2854 contigs. MTP4BH had 4608 clones with a mean size 173 kbp in the large (27.6 kbp) T-DNA vector pCLD04541, which is suitable for plant transformation and functional genomics. Plates 1–8 contained clones from the contigs belonging to the single copy regions of the genome. Plates 9 and 10 were picked from the duplicated and quadruplicated regions without redundancy, so that an individual clone represented either 2 or 4 regions per haploid genome. Plates 11 and 12 contained the marker anchored clones also used in MTP2BH. Plate 13 of MTP4BH was developed from just 6 contigs from regions with four copies by redundant picking. This set of clones should resolve into 48 regions, if methods to separate them can be developed as the genome sequencing is completed [93]. This set of 13 plates was used for HICF fingerprinting by the same methods that were used for Williams 82 [11] and PI437654 BACS [79, 96]. The BACs used for HICF will form a bridge to other physical maps and a resource to test the ability of HICF to correctly separate duplicated regions, particularly in the contigs in plate 13.

MTP4E was designed to be 4608 BAC clones with large inserts (mean 175 kbp) in the small (7.5 kbp) pECBAC1 vector [57, 85]. However, only 3840 clones were picked to date. Sequencing efficiency was low on this MTP and reracking will be needed [103]. The vector is suitable for DNA sequencing and these clones will be used for sequencing across gaps in the WGS sequence.

MTP4BH and MTP4E clones each encompassed about 800 Mbp before duplicate regions were considered. The single copy regions represented 700 Mbp [57]. In addition there were 50 Mbp from the duplicate and 50 Mbp from the quadruplicate regions in the MTP. Because those regions were duplicate and quadruplicate they encompass another 300 Mbp in total. MTP2BH, MTP4E, and MTP4BH were each used for BAC-end sequencing and microsatellite integration into the physical map [27, 39]. MTP2BH was used for EST integration to the physical map [16, 57]. MTP4BH was used for high information content fingerprinting for integration with the Williams 82 physical map [11, 104]. In conclusion, it appears like each MTP and the derived BESs will be useful to deconvolute and finish the whole genome shotgun sequence of soybean while the whole genome sequence will help complete the physical map. A complete MTP5BH would be a useful tool for functional genomics because clones from these libraries were constructed in a T-DNA vector and are ready for plant transformation. About four thousand transgenic lines made from BACs would be enough to transfer every soybean sequence to another plant.

### 4.3. BAC end sequences (BESs)

BAC end sequences (BESs) anchored to a robust physical map constitute an important tool for genome analysis, and have been developed from BACs belonging to three available MTPs including MTP2BH, MTP4BH, and MTPE4 [95, 103]. Therefore, three sets of BESs were available, of which the first set consisted of 13 474 good BESs derived from 8064 clones of MTP2BH(Table 5). Enquiries to GenBank nr and pat databases identified 7260 potentially geneic homologs, and an analysis of the locations of inferred genes suggested presence of gene-rich islands on each chromosome [37]. In addition, 42 BESs showed homology (extending over a length of 80–341 bp at e^−30^ to e^−300^) with DNA markers (10 RFLPs, 20 microsatellites) that were already genetically mapped [95]. This amounts to homology with about 2% of the markers, whose sequences are available in GenBank. Available BESs also carried as many as 1053 new SSR markers [27, 37] that are described further in the next section.

The second set of BESs consisted of 7700 good BESs reads from clones of MTP4BH (Table 5) of which 4147 had homologs in the GenBank nr and pat databases [57]. The clones in plates 11 and 12 were resequenced and so have 2 records for each BAC end in GenBank. Resequenced clones help determine the sequence error rate and greatly facilitate SNP detection [18, 19]. Twenty additional genetic anchors were detected in this second set of BESs (6 RFLPs, 14 microsatellites), which represented about 1% of the soybean markers with sequences in GenBank. This second set of BESs carried 625 SSR markers [27, 37] that are described further in the next section. The third set of BESs from MTP4E have recently been released and are only partly analyzed (Table 6).

The above builds of physical map representing recently duplicated regions of the genome can be further improved with existing databases and tools. In particular, this can be achieved by increasing the number of reliable genetic anchors derived from BESs [27, 37] and separating BACs from homoeologous regions with diagnostic SNPs (Figure 2) before contigs were formed [93].

### 4.4. Genetic map and SSR markers derived from BESs

The molecular genetic map for soybean genome can be improved further through several approaches including (i) addition of BESs markers on the available genetic map [27, 37], (ii) bioinformatics analysis of contig data [16] and (iii) through the use of novel approaches to error detection [99]. The composite genetic map of soybean at SoyGD (in 2007) contained 3073 DNA markers [16, 27], which included 1019 class I SSRs, each with >10 di- or trinucleotide repeat motifs (BARC-SSR markers; Song et al., 2004), and a few class II SSRs with <10 di- to pentanucleotide repeats that were mostly SIUC-SSR markers. Forrest BESs helped in increasing the number of class I and II SSR markers for the soybean genome, and allowed integration of BAC clones into the soybean physical map.

SSRs were mined separately from the two sets of BESs described above. As mentioned above, the first set of 10 Mbp of BAC end sequences (BESs) derived from 13 474 reads of 7050 clones constituting MTP2BH, had 1053 SSRs (333 class I + 720 from class II), and the second set of 5.7 Mbp BESs derived from 7700 reads from 5184 clones constituting MTP4BH, had 620 SSRs (150 class I + 480 class II). Potential markers are shown on the MTP_SSR track at SoyGD (Figure 6). About 530 primer pairs were designed for both the sets of SSRs. These primers were 20–24 mers long with a Tm of 55 + 1°C, and provided amplicons that were 100–500 bp long. As many as 123 of these primers belonging to duplicated regions gave multiple amplified products, and therefore should be avoided.

Different possible motifs were not randomly distributed among the above SSRs, with AT rich motifs being more frequent [27]. Compound SSRs having tetranucleotide repeats clustered with di- and trinucleotide motifs were also found. About 75% of class I and 60% of class II SSR markers were polymorphic among the parents of four recombinant inbred line (RIL) populations. Most of the BESs-SSRs were located on the soybean genetic map in regions with few BARC-SSR markers [27, 39]. Therefore, BESs-SSRs represent a useful tool for the improvement of the genetic map of soybean.

### 4.5. SNP markers derived from BESs to WGS

The soybean genome has been shown to be composed of ∼8000 short interspersed regions of one, two, or four copies per haploid genome, as shown by RFLP analysis, SSR anchors to BACs and by BAC fingerprints [16]. Recently, the genome has been sequenced by WGS sequencing of 4 kbp inserts in pUC18 [105]. When the extent and homogeneity of duplications within contigs was examined using BAC end sequences (BESs) derived from minimum tile paths (MTP2BH and MTP4BH; Figure 2) [29], a strong correlation was found between the fold of duplication inferred from fingerprinting and that inferred from WGS matches. Duplicated regions were identified by BAC fingerprint contig analysis using a criterion of less than 10% mismatch across a trace with a window size of 60 bp. Previously, simulations had predicted that fingerprints of clones from different regions would coalesce, if sequence variation was less than 2%. Hopefully, the HSVs among contigs from duplicated regions can be used to separate clone sets from different regions. Ironically, improvements for contig building methods will result from the whole genome sequence! However, many duplicated regions with less than 1% sequence divergence were found [29, 93]. The implication for bioinformatics and functional annotation of the soybean genome (and other paleopolyploid or polyploid genomes) is that reverse genetics with many genes will be nearly impossible without tools to simultaneously repress or mutate several gene family members.

## 5. FUNCTIONAL GENOMICS TOOLS

Unequivocal identification and map-based cloning of genes underlying quantitative traits have been a challenge for soybean genomics research. Gene redundancy, gene action, and low transformation efficiencies seriously hampered positional cloning [16]. Therefore, a variety of approaches need to be used for soybean functional genomics research. Two major areas of soybean genomics research include (i) annotation of genomic sequences (genes with unknown functions) and (ii) analysis of genome sequences of “Forrest” for synteny with the genomes of other dicotyledonous genera and with those of other soybean cultivars.

### 5.1. Annotation of genome sequences

The three methods that proved useful for annotation of the genome sequences of Forrest and related germplasm include (i) mutant complementation using transformation, (ii) gene silencing through RNAi, and (iii) targeted mutations. Each will be briefly discussed.

**Figure 7:**
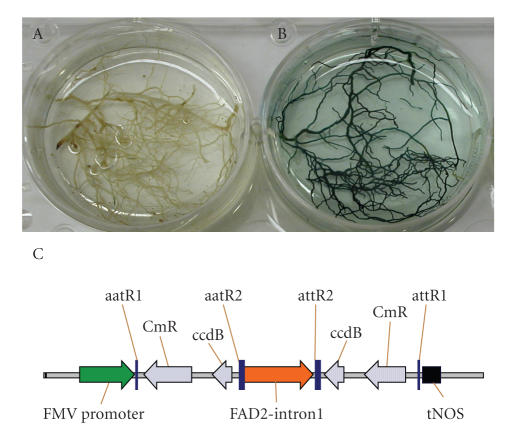
Evidence for RNAi silencing of GUS gene in 35S::GUS soybean plants. Panel A. GUS expression in composite plant roots expressing and RNAi from the gene encoding GFP. Panel B. GUS expression in composite plant roots expressing RNAi from the gene encoding GUS. Panel C. The transformation cassette used (thanks to Dr. C. G. Taylor, Danforth Center, unpublished data).

**Figure 8:**
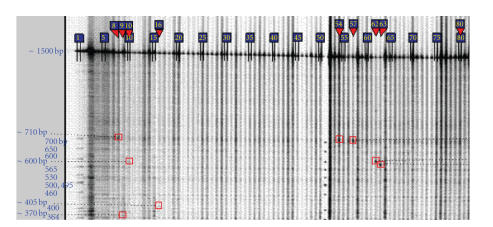
Soybean Tilling gel image of *Gm_clavata1* pool ps33 screening, representing 768 individuals, 8 individuals per pool (LI-COR 700 channel mutations are marked in red boxes; blue boxes represent lane numbers) from http://www.soybeantilling.org/ (thanks to Dr. K. Meksem and Dr. B. Liu SIUC, unpublished data).

(i) *Mutant complementation using transformation*. A popular approach for the study of gene function is mutant complementation, which involves transformation of mutants with the wild alleles. Therefore, development of transformation protocols is an essential component of functional genomics research. In soybean, *A. tumefaciens* and *A. rhizogenes*-mediated transformation of cultured cells with Forrest BAC clones has been successfully achieved using previously described protocols involving the T-DNA vector pCLD04541 [84]. In this protocol, *npt II* gene is used as a plant selectable marker, and kanamycin as used as a selective agent [106–109]. Screenable markers are available in some BAC clones (Table 7). Whole BAC transformation is important because fine maps locating loci at genetic distance of 0.25 cM that is equivalent to 50–150 kbp were earlier prepared using RILs and NILs. The clones selected for transformation are listed in Table 7, and should provide for complementation of easily scoreable phenotypes in mutants. For instance, dominant mutant phenotypes of traits like pubescence, color, and disease resistances should be evident in the very first products of transformation. BAC transformation with sets of overlapping clones will be the best approach in situations where an individual locus represents a cluster of genes [37, 38].

(ii) *Gene silencing using* *RNAi*. The composite plant system for RNAi has been tested in NILs derived from Forrest, and has been validated by Dr. C. G. Taylor at the Danforth Center (St. Louis, Mo, USA) [110] through expression of gene-specific dsRNA constructs. Using this system, shoots from stable transgenic soybean plants showing constitutive expression of *uid*A (GUS) were transformed with dsRNA constructs (Figure 7) that were designed using a modified pKannibal vector [111], with the 35S promoter replaced by the figwort mosaic virus (FMV) promoter. The 600 bp homologous sequences of the GUS or green fluorescent protein (GFP) gene were introduced in an antisense and sense orientation separated by the pKannibal intron (spacer) sequence. These constructs were designed to produce transcripts with a stem loop secondary structure that would be recognized by the plant cell machinery and activate RNAi. The dsRNA constructs placed in a binary vector, introduced into *A. rhizogenes*, were used for composite plant production [112]. GUS-specific RNAi construct silenced, while non-GUS RNAi (GFP) construct failed to silence GUS expression in hairy roots produced on shoots of transgenic soybean plants. These results show that the hairy roots can be used to produce dsRNAs. Further, the RNAi machinery in soybean hairy roots is fully functional in a sequence-specific manner. Thus, RNAi technology will allow the rapid analysis of sets of candidate genes for alleles underlying variation [38].

(iii) *Study of gene function through TILLING.* Two soybean mutagenized M2 libraries are already available for TILLING [113], from which ∼3000 of the 6000 available M2 lines were phenotyped visually. A soybean mutant database has been developed to track and sort these mutants (http://www.soybeantilling.org/). While developing a database that would allow search for “TILLED” genes a search engine was developed, so that the database can be searched for both phenotype and gene. The mutations occurred at a rate of ∼1 mutation/170 kbp, so that a screening of 6150 M2 families may provide a series of up to 40 to 60 alleles within each 1.5 kbp fragment of a target gene. This approach led to the identification of a putative mutant for a soybean leucine rich repeat receptor like kinase gene *Gm-Clavata1A* (AF197946; Figure 8). In future, TILLING and crosses among TILLED mutants [100–102] will allow the testing of candidate genes and will provide new genetic variation that may lead to germplasm enhancement.

### 5.2. Analyses of conserved synteny

Forrest genome sequences have also been used for a study of their synteny with genomes of other dicotyledonous genera/species and also with the genomes of other soybean cultivars. For this purpose, cross-species transferable genetic markers are available in the data-based legumeDB1 [114], and can be used to compare the linear order of markers/genes, which are either species specific or conserved across genera [115–124]. For instance, genes for resistance to pathogens will often appear as new genes or gene clusters inserted in regions, which otherwise exhibit conserved synteny across genera [35, 115, 122]. Synteny extends beyond genes into repeat DNA, as exemplified by the distributions of 15 bp sequences that provide sequence-specific genome fingerprints [94]. Interestingly the fingerprints do not show the same patterns of relatedness between species found in gene sequence. Therefore, genome fingerprinting will help identify good candidates for cross species markers in repeat DNA such as microsatellite markers.

Conserved synteny has also been observed among the genomes involved in the constitution of the allo- and autotetraploids hypothesized for soybean. It has been shown that about 25–30% of the genome has extensive conservation of gene order in otherwise shuffled blocks of 150–300 kbp [16]. Consequently, blocks of 3–10 genes are repeated at 2 or 4 locations per haploid genome [38, 79]. There are also genomic regions, where synteny among genomes of different cultivars has been shown to break down. Several interesting features including the following have been observed in these nonsyntenic regions: (i) in some cases, a loss of conserved synteny between cultivars is associated with a gene introgressed from a Plant Introduction [38]. (ii) In another case, a moderately repeated sequence common in one cultivar is absent in another cultivar [29]. (iii) In still another case, a sequence inserted in one cultivar appears to alter the expression of a neighboring gene (unpublished). It is thus apparent that genome analysis involving study of an association of these nonsyntenic sequence tracts in otherwise syntenic regions, with phenotypes will be an active area of research, when genome sequences from a number of soybean cultivars are available.

## 6. CONCLUSIONS

The soybean genomics resources developed through the use of cultivar Forrest have been used and will continue to be used in future leading to significant advances in soybean genomics knowledge base. The soybean genome shows evidence of a paleopolyploid origin with regions, encompassing gene-rich islands that were highly conserved following duplication [16]. In fact, it was estimated that 25–30% of the genome was highly conserved after both duplications. Implications of this feature are profound. First, a map of homoeology and an associated map of duplicated regions had to be developed. Second, an estimate of sequence conservation among the duplicated regions was necessary. Third, the implications for functional genomics were considered. Given that all soybean genes have been duplicated twice during recent evolution, and that most plant genomes encode functionally redundant pathways, it is not surprising that TILLING, RNAi-mediated silencing and overexpression of several genes often did not lead to phenotypic changes [101, 102, 110, 113]. In future, the E × F population will continue to be used for (i) an analysis of functions of a number of gene families, (ii) patenting of inventions based on useful genes [6, 77, 124–126], (iii) manipulation of soybean seed composition including protein, oil [19] and bioactive factors [127–129], and (iv) an analysis of the protein interactome [130]. In summary, the newly released E × F population and the other associated genomic resources developed through the use of cultivar “Forrest” will provide tremendous opportunities for further research in the field of genomics research.

## Figures and Tables

**Table 1:** Description of 20 linkage groups mapped in the Essex × Forrest mapping population. The map distances and markers distribution for the linkage groups were generated from analysis of the 100 F_5_-derived progeny from E × F.

		Map	No. of markers						
Linkage group	NIL^(a)^ populations	Distance (cM)	Total	SSR	RFLP	RAPD	BESs^(b)^	EST^(b)^	BESs^(c)^ SSR
A1	6	73.8	14	4	3	7	458	13	4
A2	8	259.0	22	10	8	4	757	0	7
B1	4	164.0	16	11	2	3	234	7	5
B2	5	53.4	12	7	1	4	156	3	6
C1	4	150.1	13	10	0	3	136	0	9
C2	8	213.2	30	19	4	7	565	14	4
D1a + Q	9	140.0	17	14	0	3	625	30	3
D1b+W	8	87.4	14	8	1	5	124	1	3
D2	7	245.4	19	15	0	4	122	0	4
E	6	97.4	9	6	0	3	362	11	5
F	4	219.9	29	16	5	8	369	0	2
G	12	242.5	37	19	12	6	1126	33	5
H	8	98.3	9	6	1	2	427	9	4
I	9	116.9	16	11	0	5	192	6	3
J	7	40.7	7	3	1	3	577	3	2
K	9	150.9	18	13	0	5	590	1	4
L	8	103.8	12	9	0	3	91	3	2
M	6	105.2	10	6	1	3	87	9	4
N	3	145.1	21	9	2	10	156	0	3
O	2	116.4	13	10	0	3	566	9	0
Total	100	**2823.4**	**337**	**206**	**41**	90	7720	152	79
Unlinked	(2007)	**0**	**0**	**0**	**0**	0	10529	485	10

^(a)^NIL populations segregate for 2 or more regions on different chromosomes. ^(b)^ESTs and BESs may appear at 2 or more locations on the linkage map if they appear in homoeologous regions of different linkage groups. ^(c)^BESs-SSR placedon the genetic map, many more are placed in SoyGD by inference from marker anchored contigs.

**Table 2:** Ranges and means of selected mean traits measured across multiple locations and years using the RIL population and the “Essex” and “Forrest” parents. For traits 1–35 see [24]; traits 36–79 were from [39, 42] and or unpublished.

No. of trait and symbol	Unit	RIL population	
		Average	Range
1. SDS disease incidence	Score	48.5	4.4–94
2. SDS disease severity	Score	1.5	1.1–2.3
3. SDS disease index	Score	9.3	1.1–23.9
4. Soybean cyst nematode IP	(%)	53	0–100
5. Yield during SDS	Kg*·*ha^-1^	3.3	2.9–3.76
6. Seed daidzein content	*μ*g·g^−1^	1314	874.5–2181
7. Seed genistein content	*μ*g·g^−1^	996.8	695.5–1329
8. Seed glycitein content	*μ*g·g^−1^	206.1	116–309
15. Total seed isoflavone content	*μ*g·g^−1^	2516.8	1774.2–3759
21. Resistance to manganese toxicity	Scale 0–5	2.02	1.1–4.5
32. Seed yield	Kg ha^-1^	3.44	2.64–4.13
33. Leaf trigonelline content (irrigated)	*μ*g g^−1^	98.85	59.87–126.96
34. Leaf trigonelline content (rain-fed)	*μ*g·g^−1^	417.94	245.95–618.18
35. Flower color (white: purple)	color	43:47	na
38. Mean SDS DX in Argentina	Scale 1–10	1.6	0.1–3.1
43. Tolerance to aluminum toxicity	(%)	14	−20–37
47. Seed protein content	(%)	39.5	37.5–41.5
51. Seed oil content	(%)	18.9	18.0–20.1
55. Resistance to insect herbivory (IP)	(%)	22.3	13.0–32.5
60. Seedling root growth	mm	8.3	6–11

**Table 3:** Disease resistance that segregates among the RIL and NIL population.

Disease resistance in	Causal agent
A. Forrest	
Soybean cyst nematode	*Heterodera glycines* HG type 0; races 3
Root-knot nematode	*Meloidogyne incognita*
Bacterial pustule	*Xanthomonas glycines*
Wildfire	*Pseudomonas syringae subsp. tabaci*
Target spot	*Alternaria* sp
Partial *Phytophthora* root rot	*Phytophthora sojae*
SDS root rot	*Fusarium virguliforme*
SDS leaf symptoms	Toxin

B. Essex	
Bacterial pustule	*Xanthomonas glycines*
Downy mildew	*Peronospora manshurica*
Frogeye leaf spot	*Cercospora sojina*
Purple seed stain disease	*Cercospora kikuchii*
Partial *Phytophthora* root rot	*Phytophthora sojae*
SDS leaf symptoms	Toxin

C. Hartwig	
Soybean cyst nematode	All HG Types from 1.2.3.4.5.6.7.
Root-knot nematode	*Meloidogyne incognita*
Reniform nematode	*Rotenlenchulus reniformis*
Bacterial pustule	*Xanthomonas axonopodis* pv. *glycines*
Wildfire	*Pseudomonas syringae* pv. *tabaci*
Target spot	*Corynespora cassiicol* a
Partial *Phytophthora* root rot	*Phytophthora sojae*
SDS root rot	*Fusarium virguliforme*
SDS leaf symptoms	Toxin

D. Flyer	
Powdery mildew	caused by *Microsphaera diffusa*
Purple seed strain disease	*Cercospora kikuchii*
Pod and stem blight	*Diaporthe phaselorum*
Multirace *Phytophthora* root rot	*Phytophthora sojae*
SDS leaf symptoms	Toxin

**Table 4:** Saturation mapping with markers on chromosome 18 in the 2–4 Mbp encompassing *Rhg1*, *Rfs1*, and *Rfs2* (SDS) loci with leaf and root phenotype classes shown.

Geno type	Satt214	Sat1	TMD1	Satt309	Sat185	CGG5	OI03	CTA13	Bng122	Leaf	Root
1	E	F	*E*	*E*	F	E	**F**	E	F	*S*	**R**
2	E	E	*E*	*E*	E	E	E	E	F	R	S
3	E	E	E	H	E	E	E	E	F	R	S
4	E	E	F	F	E	E	E	E	E	R	S
5	E	F	F	F	F	E	E	E	E	R	S
6	F	F	F	F	E	F	**F**	F	F	R	**R**
7	F	F	F	F	E	E	E	F	F	R	S
8	F	F	F	F	F	F	**F**	F	E	R	**R**

**Table 5:** Progress in the soybean physical map builds 2 to 5.

	Automated Build 2 Sept. 2001	Manual Build 3 Oct 2002	Manual Build 4 Oct 2003 Total	Judged by BACs/unique band to be (pploid) [unique]	Manual Build 5 Jan 2008
BAC clones in FPC database	81,024	83,026	78,001		78,001
BACs used in contig assembly	75,568	78,001	72,942		72,837
Number of singletons	5,884	4954	27,1812		17,942
Marker anchored singletons	0	0	120		63
Clone in contigs (fold genome)	69,684	73,069	45,135		58,765
Fold genome in contigs	8.7	9.1	5.6		62
Number of contigs	5,488	2,907	2,854	(646)[2208]	521
Anchoring Markers	0	385	404	(280)[124]	1,523
Anchored Contigs	0	781	742	(181)[223]	455
Contlgs contain: >25 clone	220	921	477	(268)[209]	335
10–25 clones	3,038	920	1,458	(433)[1025]	110
3–9 clones	1,845	850	820	(0)[820]	43
2 clones	385	216	99	(0)[99]	33
Unique bands in the contigs	396,843	345,457	#258,240	(64,560)	257,356
**Length of the contigs (Mb)**	1,667*	1,451*	**1.037**	**(0.258) [0.769]**	**1.034**

*Based on 4.00 kbp per unique hand. # Based on 4.05 kbp per unique band, for 2,854 contigs containing ∼68 unique bands in 15 clones, 264 duplicated region contigs containing ∼68 unique bands in 30 clones I5,840 unique bands and 406 highly repeated region contigs containing ∼68 unique bands is 60 clones, 48,720 unique bands.

**Table 6:** *Summary of sequence coverage of the three minimum tile paths (MTPs) used for BAC end sequencing made from three BAC libraries*. To calculate the percentage of the soybean genome covered by the clones (clone coverage) in our *Eco*RI-(MTP4E) and *Bam*HI or HindIII insert libraries (MTP2BH and MTP4BH), the genome size of soybean was assumed to be 1130 Mb. The BAC libraries were each constructed from DNA derived from twenty five seedlings of an inbred cultivar Forrest.

	MTP4E	MTP4BH		MTP2 BH	Totals
Vector	pBeloBAC11	pCLD04541			na
Insertion site	*Eco* RI	*Bam*HI or *Hind*III			na
Duplicates/region	1	1	2–4	1–4	na
Number of clones	3840	4608	576	8064	17 088
Mean insert size (kbp)	175 ± 7	173 ± 7	173 ± 7	140 ± 5	685
Clone coverage	0.7	0.8	0.2	1.4	3.1
BESs good reads	3 324	6772	924	13 473	25 123
BESs coverage (Mbp)	2.9	5.0	0.7	9.9	18.5

**Table 7:** Some of the BACs, mutant and nonmutant soybean lines to be transformed for complementation.

BIBAC clone names	Phenotypes	Insert size kbp	Dominant?
Gm-SIU1-B100B10	*Rhg4* bigenic resistance to SCN^(a)^	140	Yes
Gm-SIU1-B73P06	*rhg1* bigenic resistance to SCN and *Rfs2* for SDS^(a)^	79	Co-
Gm-SIU2-H050N07	*Rpg1-b* resistance to bacterial pustule^(b)^	110	Yes
Gm-SIU1-B54E07	*T* tawny pubescence; flavonoid-3-monoxygenase^(c)^	82	Yes
Gm-SIU2-H04P03	*W1* White flower and black hila color^(d)^	153	No
Gm-SIU2-H82CO8	*Rfs1* root resistance to SDS	130	Yes
Gm-SIU1-TBD	*Rps4* resistance to *Phytophthora* root rot	120	Yes

^(a)^ *Rhg4* and *rhg1* each encodes transmembrane receptor-like kinase. Resistant and susceptible alleles differ by 3–6 amino acid changes and 23 base changes. There are mutant lines derived from Forrest. ^(b)^ *Rpg1-b* encodes a nucleotide binding leucine rich repeat protein. ^(c)^ *T* encodes flanonoid-3 monoxygenase (EC1.13.14.21). The recessive genes differ from the dominant by deletion of a single C nucleotide. There are mutant lines. ^(d)^ *W* encodes an unknown enzyme, probably a glycosidase.
